# Immunohistochemical Evaluation of Androgen Receptor and Nerve Structure Density in Human Prepuce from Patients with Persistent Sexual Side Effects after Finasteride Use for Androgenetic Alopecia

**DOI:** 10.1371/journal.pone.0100237

**Published:** 2014-06-24

**Authors:** Carla Di Loreto, Francesco La Marra, Giorgio Mazzon, Emanuele Belgrano, Carlo Trombetta, Sabina Cauci

**Affiliations:** 1 Department of Medical and Biological Sciences, School of Medicine, University of Udine, Udine, Italy; 2 Urological Hospital Department, Department of Medical, Surgical and Health Sciences, University of Trieste, Trieste, Italy; H. Lee Moffitt Cancer Center & Research Institute, United States of America

## Abstract

Finasteride is an inhibitor of 5-α-reductase used against male androgenetic alopecia (AGA). Reported side effects of finasteride comprise sexual dysfunction including erectile dysfunction, male infertility, and loss of libido. Recently these effects were described as persistent in some subjects. Molecular events inducing persistent adverse sexual symptoms are unexplored. This study was designed as a retrospective case-control study to assess if androgen receptor (AR) and nerve density in foreskin prepuce specimens were associated with persistent sexual side effects including loss of sensitivity in the genital area due to former finasteride use against AGA. Cases were 8 males (aged 29–43 years) reporting sexual side effects including loss of penis sensitivity over 6 months after discontinuation of finasteride who were interviewed and clinically visited. After informed consent they were invited to undergo a small excision of skin from prepuce. Controls were 11 otherwise healthy matched men (aged 23–49 years) who undergone circumcision for phimosis, and who never took finasteride or analogues. Differences in AR expression and nerve density in different portions of dermal prepuce were evaluated in the 2 groups. Density of nuclear AR in stromal and epithelial cells was higher in cases (mean 40.0%, and 80.6% of positive cells, respectively) than controls (mean 23.4%, and 65.0% of positive cells, respectively), P = 0.023 and P = 0.043, respectively. Conversely, percentage of vessel smooth muscle cells positive for AR and density of nerves were similar in the 2 groups. The ratio of AR positive stromal cells % to serum testosterone concentrations was 2-fold higher in cases than in controls (P = 0.001). Our findings revealed that modulation of local AR levels might be implicated in long-term side effects of finasteride use. This provides the first evidence of a molecular objective difference between patients with long-term adverse sexual effects after finasteride use versus drug untreated healthy controls in certain tissues.

## Introduction

Finasteride is a synthetic 4-azasteroid molecule, used in the treatment of both androgenetic alopecia (AGA) and benign prostatic hyperplasia (BPH) [1]. Finasteride impedes the *in loco* conversion of testosterone (T) into dihydrotestosterone (DHT) by inhibiting the 5-α-reductase (5-α-R) enzyme, primarily the 5-α-R type 2, in tissues and liver [1]–[3]. In adults, DHT is known to act as primary androgen in prostate and hair follicles [1], [2], [4].

Androgens, in association with genetic factors, have been involved in AGA pathogenesis [5], [6]. Circulating and local androgen hormones are considered a necessary (although not sufficient) condition for the development of AGA. T and DHT were demonstrated to induce apoptosis in dermal papilla cells (DPC) in a dose-dependent and time-related manner [7]. In the majority of men with male pattern baldness endogenous production of DHT is markedly increased, providing a rationale for therapeutic 5 alpha-reductase inhibition in this disorder [8].

The molecular action of androgens is mediated by the androgen receptor (AR), a member of steroid hormone receptors family [9], [10]. Testosterone or DHT binds to AR present in the cell cytoplasm, the ligand binding activates the receptor to translocate into the nucleus, homodimerize, and act as a transcription factor binding specific DNA response elements called AREs (androgen response elements) present in different target genes to mostly up-regulate gene expression [4]. Thus, AR is responsible for androgens physiological effects through activation of specific androgen responsive genes [11]. Presently, it is questioned whether blocking of T conversion to DHT by finasteride in prostate tissue will change expression of AR [12].

According to the recent Mella and colleagues meta-analysis [13], there is moderate quality evidence that inhibition of DHT synthesis by finasteride (usually at a dosage of 1 mg/day) is able to prevent AGA progression and to increase hair density and quality in male patients [13]. The main limit of finasteride use against AGA is the onset of side effects [14]. More common adverse side effects are: erectile dysfunction, loss of libido and ejaculation disorders [13], [15]. Less common side effects include anxiety, depression, gynecomastia, and breast cancer in men [16]. Of concern, recently, it has been reported that adverse side effects of finasteride used to treat AGA might be persistent several months or years after finasteride discontinuation [14], [17]–[19]. The actual incidence of this phenomenon is still to be determined, but such negative events impairing sexual health in young males causes a severe loss in terms of life quality [14], [17]. In year 2011 a study by Irwig and Kolukula [18] first characterized the types and duration of persistent sexual side effects that occurred in 71 otherwise healthy men during or immediately after taking finasteride for the treatment of AGA. Most men developed sexual dysfunction in multiple domains with 94% experiencing low libido, 92% experiencing erectile dysfunction, 92% experiencing decreased arousal, and 69% experiencing problems with orgasm [18].

Since June 2010 the pharmaceutical company (Merck) that produces Propecia (finasteride 1 mg/day for AGA treatment) included in the drug sheet as rare but possible side effects erection disorders persistent after drug discontinuation, and infertility. In April 2012, the US Food and Drug Administration required to expand the list of persistent sexual adverse events indicated in the labels for Propecia [16]. Of interest, many patients with long-term adverse effects refer of skin discomfort in genital area; severity of this effect in some cases is even described as a local paresthesia. The origin of these phenomena is not known.

In the present study, we investigated the reasons of reduced or altered sensitivity localized in the genital area in 8 men who took finasteride to treat AGA and who also suffered from sexual dysfunction including discomfort, numbness or even paresthesia of the skin in the genital area, persistent loss of libido and erectile dysfunction over 6 months after discontinuing finasteride treatment. To assess the sensitivity issue we collected foreskin samples from all case patients to determine nerve density and we compared the results with similar samples from 11 age-matched healthy finasteride untreated controls that underwent circumcision to treat phimosis. We also compared the AR tissue expression between the 2 groups and explored the relation between AR and the symptoms showed by the case patients.

## Patients and Methods

### Ethics Statement

Enrolment and medical visits of all patients were performed at the Urological Unit of University Hospital of Trieste, whereas diagnostic analyses were carried out according to routine laboratory procedures at the University Hospital of Udine. The Institutional Ethical Committee of each participating institution approved the study protocol, and all the subjects signed a written informed consent before entering the study. The study was conducted according to the principles expressed in the Declaration of Helsinki.

### Participants

The study design was a retrospective case-control study. All participants were white Caucasian males. Exclusion criteria for both cases and controls were presence of obesity (BMI >30 kg/m^2^), any acute or chronic disease like diabetes mellitus, hypertension, cardiovascular diseases, thyroid diseases, autoimmune pathologies and malignancies.

Cases were 8 patients enrolled after clinical consult (n = 5) or through Propeciahelp.com (n = 3), a forum collecting reports and experiences of patients affected by persistent sexual side effects after finasteride discontinuation. Eligibility criteria were: history of finasteride use for AGA, sexual side effects including self-reported loss of sensitivity in the genital area arose during or soon after finasteride treatment and lasting 6 or more months after drug discontinuation. Patients suffering from sexual disorders before finasteride use, or who had any hormonal supplementation in the last 6 months were excluded. All former finasteride users were otherwise healthy, 2 of them were subsequently enrolled also into the study by Melcangi and colleagues, for evaluation of steroid levels in cerebrospinal fluid [20].

Eleven controls were enrolled among subjects undergoing circumcision because of phimosis. Eligibility criteria for controls were: healthy, AGA positive, with no history of finasteride or any other drug capable of impairing androgens action use (like dutasteride, saw palmetto, antidepressants, GnRH agonists, isotretionin), and no use of hormonal treatments ever. Subjects with any sexual dysfunction and/or chronic or acute diseases with exception of phimosis were excluded from controls. Enrolled subjects, i. e. both cases and controls, were evaluated for presence of AGA by the Hamilton-Norwood scale through a questionnaire reporting the pictures showing grades from 1 to 7; all enrolled subjects had grade 2 or more [21].

Two different structured questionnaires were administered to the former finasteride users (cases) to evaluate the development and severity of persistent side effects. An ad-hoc questionnaire was elaborated by the study authors to interview cases about demographic and clinical characteristics, lifestyle habits, finasteride dosage, period of drug use, onset, type and duration of side-effects. The duration of the finasteride therapy was calculated in days of use. In particular, we asked patients to report in detail every persistent symptom started during and/or soon after taking finasteride and never experienced before finasteride use. We asked also to provide a copy of any diagnostic exam performed in their life. It is to note that all of them had some laboratory exams performed after finasteride use, whereas none subject, except 1, had any kind of laboratory exams performed before finasteride use. No subject had alterations of liver or thyroid function tests. 

Additionally, formers finasteride users filled the Arizona Sexual Experience Scale (ASEX) questionnaire [22]. The ASEX questionnaire was chosen as validated instrument to assess sexual function before and after taking finasteride. Possible total ASEX scores range from 5 to 30, with the higher scores indicating more severe sexual dysfunction. Sexual dysfunction is considered to be present if the total score is ≥19 or if any one item is ≥5 or if any three items are ≥4. We asked patients to complete ASEX citing their symptoms at the moment of interview. Additionally, we asked them to fill the ASEX questionnaire referring their condition before taking finasteride.

Designing the study we observed that not all patients had finasteride at the dosage exactly of 1 mg/day (Propecia dosage), many patients used the dosage of 1.25 mg/day because they preferred to buy the less costly 5 mg finasteride pills (Proscar) and then to break them into four parts (of 1.25 mg each). For this reason we decided to consider all of them.

### Histological specimens

Prepuce samples were acquired from cases through punch biopsy; a 5 mm square surface of skin, 2 to 4 mm deep was acquired, while specimens of controls was obtained from circumcision tissue. All specimens were retrieved by one physician. Buffered formalin at 4% concentration was used to fix samples. Both a hematoxylin and eosin stain and an immunohistochemistry slides were acquired for all samples. The primary antibody used to mark the AR was Androgen Receptor Clone AR 441, murine monoclonal M3562 (Dako A/S, Denmark). To obtain immunohistochemistry slides Autostainer Ling 48 was used (Dako A/S, Denmark). We used Envision+/HRP rabbit/mouse polymer as detection system. We then performed through light microscopy a semi-quantitative evaluation of AR expression in epithelial, stromal and vessel smooth muscle cells. Percent of cells of each type positive for nuclear AR were reported.

We also estimated nerve density on hematoxylin and eosin stained slides. For this purpose, each nerve seen in the slide was counted as one. All the reported figures were the average of 25 different fields at 20× magnification. All evaluations were performed by an expert physicians blinded to subjects characteristics.

Testosterone was measured in the venous blood serum by competitive chemiluminometric immunoassay on the automated Advia Centaur CP-Siemens Healthcare analyzer with reference interval 8.4–28.7 nmol/L, free testosterone was calculated by Vermuelen et al. 1999 method [23], reference interval was 200–800 pmol/L.

### Statistical analysis

Continuous data were expressed as mean and standard deviation. Mann–Whitney U test was used to compare results from continuous variables. Fisher's test was used to compare results from categorical variables. Two-sided Spearman's Rho coefficient was used to assess correlations of continuous values. Two-sided P values <0.05 were considered significant. Statistical analyses were performed by SPSS (Statistical Package for Social Sciences) software.

## Results

Characteristics of 8 cases (former finasteride users having long-lasting sexual side effects) along with results from the questionnaires and clinical visit, were summarized in Table 1. Patients self reported shrinkage of the penis. Former finasteride users were 29–43 years old, all were single, none declared himself as homosexual or bisexual. Patients started finasteride use in the age range of 22–32 years, half took finasteride 1 mg/day, and half had 1.25 mg/day. All these patients spontaneously declared that finasteride use effectively stopped their hair loss. Finasteride was assumed on average for nearly 32 months (i. e. almost 3 years), with a range from 49 to 3100 days. In all subjects adverse side effects persisted for over 6 months after finasteride discontinuation and were still affecting the patients at the time of the visit day for study enrolment. Patients were enrolled on average at nearly 56 months (i.e. almost 5 years), with a range from 240 to 3010 days after drug discontinuation. All patients claimed insensitivity in the genital area and had ASEX score ≥19 indicating sexual dysfunction. In particular, all of the 8 patients had loss of penis sensitivity and loss of pleasurable response to touch, 7 out of 8 cases (87.5%) had loss of scrotum and/or testicles sensitivity, hardened tissue and/or rubbery texture, flaccidity, wrinkledness and retraction into the scrotum, and erectile dysfunction. All cases except 2 reported reduced penis dimension and declared pain in penis and/or scroto or testis. Collection of medical history data revealed that 1 patient had a documented history of low serum total testosterone and 4 had a documented history of low serum free testosterone after finasteride use but before present study entry. Neither patient measured serum hormone levels, nor had health provider consultation for any sexual symptom before finasteride use. None patient declared use of drugs to augment sexual functions before finasteride use, whereas 5 used tadalafil (Cialis) after finasteride discontinuation.

**Table 1:** Characteristics of 8 patients former finasteride users, finasteride self-reported side effects and sexual function information.

Variable	8 Cases n (%) or mean ± SD	P value^a^
Finasteride dose (1 mg per day), n (%)	4 (50.0)	
Finasteride dose (1.25 mg per day), n (%)	4 (50.0)	
Age at starting finasteride use (y)	28.5±3.34	
Duration of finasteride use (days)	956±1130	
Discontinuation of finasteride (days)	1672±862	
Present ASEX score (points)	22.5±2.78	
Pre-finasteride use ASEX score (points)	7.6±1.92	**<0.001** a
Loss of penis sensitivity, n (%)	8 (100)	
Loss of penis pleasurable response to touch, n (%)	8 (100)	
Loss of scrotum and/or testicles sensitivity, n (%)	7 (87.5)	
Hardened tissue and/or rubbery texture, n (%)	7 (87.5)	
Flaccidity, wrinkledness and retraction into the scrotum, n (%)	7 (87.5)	
Erectile dysfunction, n (%)	7 (87.5)	
Pain in penis and/or scroto or testis, n (%)	6 (75.0)	
Reduced penis dimension, n (%)	6 (75.0)	
Reduced scroto or testis dimension, n (%)	5 (62.5)	
Reduced ejaculate volume, n (%)	5 (62.5)	
Reduced pubic hair, n (%)	4 (50.0)	
History of low total testosterone detected once after finasteride discontinuation and before present study entry	1 (12.5)	
History of low free testosterone detected once after finasteride discontinuation and before present study entry	4 (50.0)	
Tadalafil (Cialis) post-finasteride use, n (%)	5 (62.5)	
Use of testosterone supplementation after finasteride discontinuation,^b^ n (%)	3 (37.5)	
Use of benzodiazepines after finasteride discontinuation, n (%)	2 (25.0)	

a Comparison between present and pre-finasteride use ASEX scores, 2-sided P value was evaluated by the Mann-Whitney U-test.

b Testosterone supplementation post-finasteride was terminated over 1 year before present study entry.

Comparison of characteristics, immunohistochemical and serum total and free testosterone levels in cases and controls were illustrated in Table 2. Controls were 23–49 years old, they did not differ in age and BMI from cases. Controls were more frequently married than cases (P = 0.045), none declared himself as homosexual or bisexual, they had no sexual disturbance except discomfort due to phimosis; none declared use of drugs to ameliorate erectile functions.

**Table 2:** Comparison of demographics features, immunohistochemical findings and serum hormonal levels between 8 cases and 11 controls.

Variable	Cases n = 8	Controls n = 11	P value^a^
Age (y)	36±4.7	34±8.9	0.710
BMI (kg·m^−2^)	23.5±2.28	24.6±2.71	0.364
University Education, n (%)	7 (87.5)	7 (63.6)	0.338
High school, n (%)	1 (12.5)	4 (36.4)	0.338
Married or stable partner, n (%)	0 (0)	5 (45.5)	**0.045**
Smoker, n (%)	3 (37.5)	5 (45.5)	1.000
Nuclear AR positive epithelial cells (%)	80.6±8.63	65.0±19.10	**0.043**
Nuclear AR positive stromal cells (%)	40.0±15.1	23.4±8.68	**0.023**
Nuclear AR positive vessel smooth muscle cells (%)	3.13±3.04	3.41±2.61	0.331
Average tissue AR positive cells (%)	41.2±4.46	30.6±8.75	**0.007**
Nerves density (%)	2.22±0.653	1.91±0.952	0.215
Total testosterone (nmol/L)	11.6±2.96	13.6±3.45	0.290
Total testosterone below 8.4 nmol/L, n (%)	1 (12.5)	0 (0)	0.421
Free testosterone (calculated) (pmol/L)	218±52.2	270±80.2	0.413
Free testosterone (calculated) below 200 pmol/L, n (%)	2 (25.0)	0 (0)	0.164
Ratio of AR positive stromal cells (%) and serum total testosterone (nmol/L)	3.47±1.25	1.86±0.372	**0.001**
Ratio of AR positive stromal cells (%) and serum free testosterone (pmol/L)	0.192±0.095	0.096±0.027	**0.005**

a Two-sided P values to assess differences of continuous variables and of categorical variables were tested by means of the Mann-Whitney U-test or the Fisher's exact test, as appropriate.

**Figure 1:**
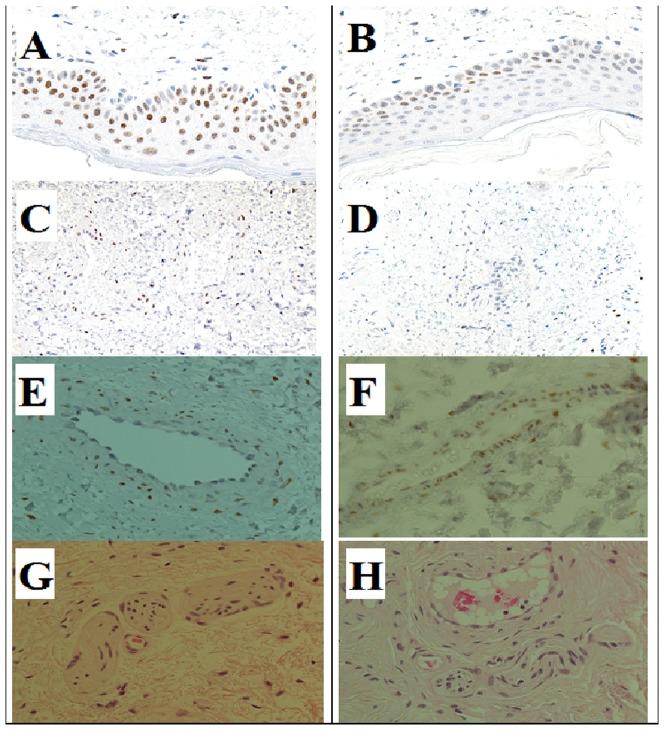
AR positive cells (by brown staining): (A) case, (B) control in epithelial cells; (C) case, (D) control in stromal cells; (E) case, (F) control in vessels smooth muscle cells. Nerves in foreskin tissue from a case (G) and a control (H).

The percentage of cells positive to AR in the nuclei of epithelial, stromal and vessel smooth muscle cells, were evaluated by immunohistochemistry, examples were reported in Figure 1, panels from A to F. Percentage of epithelial cells (basal cells of the epidermis) positive for nuclear AR was higher in cases (mean±SD, 80.6±8.63%) than in controls (mean±SD, 65.0±19.1%), P = 0.043. Stromal cells in cases showed an almost 2-fold greater expression of AR in the nuclei compared to controls (mean±SD, 40.0±15.1% in cases versus 23.4±8.68% in controls), P = 0.023. Percentage of AR positive vessel smooth muscle cells did not differ between the 2 groups. The average of AR positive cells in the 3 kind of tissues was higher in cases than in controls, P = 0.007 (Table 2).

Panels G and H in Figure 1 show representative pictures of nerve densities in a case (Panel G) and a control (Panel H) subject. Histological evaluation of nerve density revealed that patients showed similar density compared to controls (Table 2).

Serum total testosterone concentration was slightly lower, but not significant different, in cases than in controls. Similarly, free T (calculated according to [23]) concentrations were slightly lower, but not significant different, in cases compared to controls. Only 1 case but none control had total T concentration lower than normal value (8.4 nmol/L). Moreover, 2 cases but none control had free T concentration lower than normal value (200 pmol/L).

To roughly estimate the effect of circulating testosterone on local AR expression we evaluated the ratio of immunohistochemically determined AR in tissues and serum total T or free T. Interestingly, as reported in Table 2, the ratio of AR positive stromal cells (%) and serum T was 2-fold higher in patients than in controls (P = 0.001). Similarly, the ratio of AR positive stromal cells (%) and serum free T was 2-fold higher in patients than in controls (P = 0.005).

Analysis of bivariate correlations between sexual dysfunction in former finasteride users as described by the ASEX score points and laboratory biomarkers continuous values highlighted that present ASEX points were inversely related to % of AR positive stromal cells (Rho  = −0.722, P = 0.043). This effect was even stronger considering as variable the difference between present and pre-finasteride use ASEX score in relation to AR in stromal cells (Rho  = −0.913, P = 0.002). Contrariwise, ASEX score before finasteride use was not correlated to AR in stromal cells (Rho  = 0.118, P = 0.781).

Finally, the ratios of AR positive stromal cell (%) over total T concentrations and over free T concentrations had a strong inverse relationship with the variable ASEX difference (present minus pre-finasteride ASEX points), Rho  = −0.988, P<0.001; and Rho  = −0.855, P = 0.007, respectively.

## Discussion

Finasteride is still one of the most common therapeutic drugs prescribed for AGA, a distinctive alopecia pattern involving hairline recession and vertex balding, a condition more frequent with increasing age [24]–[26]. An European study found moderate to extensive male pattern hair loss in approximately 12% of men aged 18 to 40 years [27]. Finasteride therapy against AGA (at 1 mg/die, for 3 months or longer) is usually given to younger men than against prostatic disorders (at 5 mg/die) [28], [29]. Finasteride was proposed for treatment of adolescent androgenic alopecia [30], and hidradenitis suppurativa in children and adolescent [31]. However, treatment of young subjects is of increasing concern due to accumulating evidence that daily use of oral finasteride has several severe adverse effects [13], [14], [17]–[19].

Our main finding was the assessment of a significant increase of AR nuclear levels in some types of cells, specifically, stromal and epithelial cells, in dermis samples of foreskin from patients with major sexual adverse side-effects long after use of finasteride (on average almost 5 years later). As our patients are suffering from symptoms suggestive of local androgens deficiency, it was important to assess if this phenomenon was due to intrinsic inability to express and/or translocate the AR into the cell nuclei, specifically in the genital tissues. Our present data permit to exclude an impairment of this kind, at least in the foreskin. It is to note also that our data indicate that AR levels in nuclei of vessel muscle cells were similar in cases and controls, thus the increase in AR observed in former finasteride users was tissue specific. Our observation is apparently in line with an immunohistochemical study performed on 47 cases of BPH treated with finasteride (5 mg/die) analyzed before transurethral resection of the prostate, which showed significant upregulation of ARs by finasteride treatment for 30–180 days [12]. Additionally, in an androgen-dependent prostate cancer cell line (LNCaP) cells treated by finasteride significant *in vitro* upregulation of ARs was demonstrated [12], [32].

The unexpected result of our present study is that the AR upregulation was detectable in the dermis of subjects long after discontinuation of finasteride use, on average at almost 5 years (mean 1672 days) of drug wash-out, in a range from 8 months to over 8 years (240 to 3010 days). Contrariwise, the hormonal alteration effects of finasteride have been reported to be shortly reversible. In fact, according to drug information provided by Lexi-Comp., after 6 months of treatment with 5 mg/day finasteride the circulating DHT levels are reduced to castrate levels without significant effects on circulating testosterone and levels return to normal within 14 days of discontinuation of treatment [16].

An intriguing observation of our study was that the ratio of AR positive stromal cells (%) to serum total or free testosterone was 2-fold higher in former finasteride users than controls. This finding could indicate the presence in former finasteride users of an augmented regulatory feedback loop that normally serves to modulate hormonal responses [33]–[35]. Such amplification of AR levels in respect to serum testosterone levels could derive from hormonal ipo-response in the genital tissues. However, we cannot presently exclude that this apparent upregulation of AR expression is due to local low levels of androgens (particularly DHT) [36]. Indeed, it is known that androgens themselves can regulate AR transcription both in a positive and a negative way. T and DHT may down-regulate AR mRNA by decreasing its transcription; on the other hand androgens can increase AR half life by stabilizing the receptor in its dimer form [33], [34]. In addition, finasteride can directly bind to AR just as T, thus, finasteride may act directly as a competitor to androgens and exert an inhibiting role. Finasteride effects as an inhibitor are reported as light but stable [37].

In general, the AR levels can be modulated by several factors [38], [39]. Our unexpected observation that percentage of AR positive stromal cells is inversely related to ASEX score, suggests that patients less able to raise AR are those with more severe side effects related to sexual dysfunction. This seems to support the hypothesis that the body tries to compensate local androgens deprivation by producing more androgen receptors. Notably, 2 of our patients were found to have low androgen levels in the cerebrospinal fluids by Melcangi et al. [20]. Further studies are needed to assess these complex issues. The design of our study does not allow determining whether the AR in the nuclei of patients is indeed able to properly act as transcription factor like in healthy subjects. It cannot be excluded, for example, that epigenetic changes [39]–[45] induced by finasteride use could have modified the transcriptional activity of AR present in the nuclei, which is potentially able to modulate around 500 AREs and 200 AR responsive genes [46].

Most of our patients suffered erection disorders. Erection is a complex process involving androgens actions and also important signaling in the brain [47]–[49]. It is generally accepted that androgens are really important in masculine behaviour, although the relation between testosterone concentrations and sexual behaviour is not well understood. Some authors [50] suggested a possible mechanism for lack of androgens induced by 5-α-R inhibitor therapy to cause erectile dysfunction. Hypoandrogenism is believed to cause corpora cavernosa fibrosis through collagen fibres deposition and inhibition of nitric oxide synthases [50], [51]. Moreover, Zhang and colleagues [52] demonstrated that 5-α-R inhibitor therapy attenuates erectile function by promoting apoptosis in the cavernous smooth muscle cells of aged rats, suggesting a new role for androgen in maintaining the structural and functional integrity of the erectile organ.

Since finasteride inhibits T conversion into DHT, which is responsible for most androgen activity, it is plausible that prolonged finasteride use in predisposed individuals could simulate the effects of aging in young men. Since some of the effects of androgen inhibition can not be reversed once local androgen levels are re-established, it is temping to speculate that patients could still suffer from adverse sexual effects several months or even permanently after finasteride discontinuation because of aging effects caused prematurely by androgens deprivation, namely by artificially reduced DHT concentrations. Indeed, 2 of our patients were found to have low DHT levels in the cerebrospinal fluids long after finasteride discontinuation by Melcangi et al. [20].

Our study has several limitations, first limitation is the small number of subjects enrolled, which is derived in part by the strict selection of patients, which had to show severe side-effects including specifically loss of sensitivity in the genital area persistent for more than 6 months after drug discontinuation, a symptom that had to be totally absent before finasteride use.

A second limitation is due to the impossibility to determine DHT levels, especially locally, which could help in interpretation of results. Finasteride is a drug which specifically dampens DHT levels [53]. DHT has a 10-fold higher potency of inducing AR signalling than T, and reduction of DHT and neurosteroids by finasteride may influence sexual behavior [14], [54]. In fact, it was demonstrated that in patients with male-pattern baldness treatment with finasteride is effective in reducing local DHT/T in scalp hair [5]. Consistently, DHT was formed in negligible amounts in fibroblasts cultured from the genital skin of a patient with 5 alpha-reductase deficiency [55].

A third limitation of our study was the absence of genetics evaluation of polymorphisms in the AR gene, that can potentially be responsible for alterations in AR expression, and which can also determine AGA [56]. However, in part, we avoided this confounding by enrolling matched controls with levels of AGA similar to those declared by patients before assuming finasteride. Indeed, a very recent paper demonstrated no difference in AR polymorphisms between patients with post-finasteride syndrome (PFS) and untreated controls having AGA [57].

Finally, an obvious limitation of our study is due to the retrospective design, thus, we had no samples available for measurements from patients before and/or during finasteride use.

On the other hand, strength of our study is that we documented severe sexual side-effects in patients who were carefully clinically examined by an expert andrologist. It is to note that other studies on long term side-effects of finasteride used against AGA collected information by internet or phone call on subjects that self-reported symptoms [17], [18].

An important issue related to loss of sensitivity in the penis and scrotal/testicular area was to explore if this condition was the consequence of an alteration in nerve density. We demonstrated that cases and controls had similar nerve densities, thus no structural nerve alteration seems responsible for this kind of finasteride side-effect. Interestingly, in a rat model, finasteride treatment for 4 weeks reduced the weight of the corpus cavernosum but appears not to affect the erectile responses to electrical stimulation of the cavernous nerve [49]. The long-term effects of finasteride on both central and peripheral neural pathways of erection will require further investigations. In fact, it is known that androgens and AR have roles in regrowth of peripheral nerves and AR is also expressed in the central nervous system [40], [58]–[60].

Our study was the first to document an alteration of AR levels in genital tissues from young patients with long-term adverse sexual effects after finasteride use against AGA. Further enlarged studies will be necessary to better assess which are the causative factors of persistent sexual side effects observed in some men who used finasteride against AGA. A better knowledge of molecular events occurring during and/or after finasteride use could suggest possible remedies against severe sexual side-effects in fertile age young men.

## References

[1] Rittmaster RS (1994) Finasteride. N Engl J Med 330: 120–125. 7505051 10.1056/NEJM199401133300208

[2] Anitha B, Inamadar AC, Ragunatha S (2009) Finasteride-its impact on sexual function and prostate cancer. J Cutan Aesthet Surg 2: 12–16. 20300365 10.4103/0974-2077.53093 PMC2840927

[3] Traish AM (2012) 5alpha-reductases in human physiology: an unfolding story. Endocr Pract 18: 965–975. 23246684 10.4158/EP12108.RA

[4] Lee HJ, Chang C (2003) Recent advances in androgen receptor action. Cell Mol Life Sci 60: 1613–1622. 14504652 10.1007/s00018-003-2309-3 PMC11146054

[5] Ryu HK, Kim KM, Yoo EA, Sim WY, Chung BC (2006) Evaluation of androgens in the scalp hair and plasma of patients with male-pattern baldness before and after finasteride administration. Br J Dermatol 154: 730–734. 16536818 10.1111/j.1365-2133.2005.07072.x

[6] Yip L, Rufaut N, Sinclair R (2011) Role of genetics and sex steroid hormones in male androgenetic alopecia and female pattern hair loss: an update of what we now know. Australas J Dermatol 52: 81–88. 21605090 10.1111/j.1440-0960.2011.00745.x

[7] Winiarska A, Mandt N, Kamp H, Hossini A, Seltmann H, et al (2006) Effect of 5alpha-dihydrotestosterone and testosterone on apoptosis in human dermal papilla cells. Skin Pharmacol Physiol 19: 311–321. 16931898 10.1159/000095251

[8] Vierhapper H, Nowotny P, Maier H, Waldhausl W (2001) Production rates of dihydrotestosterone in healthy men and women and in men with male pattern baldness: determination by stable isotope/dilution and mass spectrometry. J Clin Endocrinol Metab 86: 5762–5764. 11739436 10.1210/jcem.86.12.8078

[9] Aranda A, Pascual A (2001) Nuclear hormone receptors and gene expression. Physiol Rev 81: 1269–1304. 11427696 10.1152/physrev.2001.81.3.1269

[10] Huang P, Chandra V, Rastinejad F (2010) Structural overview of the nuclear receptor superfamily: insights into physiology and therapeutics. Annu Rev Physiol 72: 247–272. 20148675 10.1146/annurev-physiol-021909-135917 PMC3677810

[11] Patrao MT, Silva EJ, Avellar MC (2009) Androgens and the male reproductive tract: an overview of classical roles and current perspectives. Arq Bras Endocrinol Metabol 53: 934–945. 20126845 10.1590/s0004-27302009000800006

[12] Hsieh JT, Chen SC, Yu HJ, Chang HC (2011) Finasteride upregulates expression of androgen receptor in hyperplastic prostate and LNCaP cells: Implications for chemoprevention of prostate cancer. Prostate 71: 1115–1121. 21557276 10.1002/pros.21325

[13] Mella JM, Perret MC, Manzotti M, Catalano HN, Guyatt G (2010) Efficacy and safety of finasteride therapy for androgenetic alopecia: a systematic review. Arch Dermatol 146: 1141–1150. 20956649 10.1001/archdermatol.2010.256

[14] Traish AM, Hassani J, Guay AT, Zitzmann M, Hansen ML (2011) Adverse side effects of 5alpha-reductase inhibitors therapy: persistent diminished libido and erectile dysfunction and depression in a subset of patients. J Sex Med 8: 872–884. 21176115 10.1111/j.1743-6109.2010.02157.x

[15] Nickel JC, Fradet Y, Boake RC, Pommerville PJ, Perreault JP, et al (1996) Efficacy and safety of finasteride therapy for benign prostatic hyperplasia: results of a 2-year randomized controlled trial (the PROSPECT study). PROscar Safety Plus Efficacy Canadian Two year Study. CMAJ 155: 1251–1259. 8911291 PMC1335066

[16] Finasteride (2014) Drug information provided by Lexi-Comp. Merck Manuals Online Medical Library for Healthcare Professionals http://www.merck.com/mmpe/print/lexicomp/finasteride.html. accessed April, 2014.

[17] Irwig MS (2012) Persistent sexual side effects of finasteride: could they be permanent? J Sex Med 9: 2927–2932. 22789024 10.1111/j.1743-6109.2012.02846.x

[18] Irwig MS, Kolukula S (2011) Persistent sexual side effects of finasteride for male pattern hair loss. J Sex Med 8: 1747–1753. 21418145 10.1111/j.1743-6109.2011.02255.x

[19] La Marra F, Di Loreto C, Mazzon G, Chiriacò G, Trombetta C, Cauci S (2012) Preliminary evidence of a peculiar hormonal profile in men with adverse effects after use of finasteride against androgenetic alopecia. Am J Pathol 181: S8.

[20] Melcangi RC, Caruso D, Abbiati F, Giatti S, Calabrese D, et al (2013) Neuroactive Steroid Levels are Modified in Cerebrospinal Fluid and Plasma of Post-Finasteride Patients Showing Persistent Sexual Side Effects and Anxious/Depressive Symptomatology. J Sex Med 10: 2598–2603. 23890183 10.1111/jsm.12269

[21] Norwood OT (1975) Male pattern baldness: classification and incidence. South Med J 68: 1359–1365. 1188424 10.1097/00007611-197511000-00009

[22] McGahuey CA, Gelenberg AJ, Laukes CA, Moreno FA, Delgado PL, et al (2000) The Arizona Sexual Experience Scale (ASEX): reliability and validity. J Sex Marital Ther 26: 25–40. 10693114 10.1080/009262300278623

[23] Vermeulen A, Verdonck L, Kaufman JM (1999) A critical evaluation of simple methods for the estimation of free testosterone in serum. J Clin Endocrinol Metab 84: 3666–3672. 10523012 10.1210/jcem.84.10.6079

[24] Otberg N, Finner AM, Shapiro J (2007) Androgenetic alopecia. Endocrinol Metab Clin North Am 36: 379–398. 17543725 10.1016/j.ecl.2007.03.004

[25] Rathnayake D, Sinclair R (2010) Male androgenetic alopecia. Expert Opin Pharmacother 11: 1295–1304. 20426708 10.1517/14656561003752730

[26] Rhodes T, Girman CJ, Savin RC, Kaufman KD, Guo S, et al (1998) Prevalence of male pattern hair loss in 18–49 year old men. Dermatol Surg 24: 1330–1332. 9865198 10.1111/j.1524-4725.1998.tb00009.x

[27] Budd D, Himmelberger D, Rhodes T, Cash TE, Girman CJ (2000) The effects of hair loss in European men: a survey in four countries. Eur J Dermatol 10: 122–127. 10694311

[28] Kaufman KD (2002) Androgens and alopecia. Mol Cell Endocrinol 198: 89–95. 12573818 10.1016/s0303-7207(02)00372-6

[29] Group. FMPHLS (2002) Long-term (5-year) multinational experience with finasteride 1 mg in the treatment of men with androgenetic alopecia. Eur J Dermatol 12: 38–49. 11809594

[30] McDonough PH, Schwartz RA (2011) Adolescent androgenic alopecia. Cutis 88: 165–168. 22106721

[31] Randhawa HK, Hamilton J, Pope E (2013) Finasteride for the treatment of hidradenitis suppurativa in children and adolescents. JAMA Dermatol 149: 732–735. 23552442 10.1001/jamadermatol.2013.2874

[32] Friedman AE (2012) Comment on "Finasteride upregulates expression of androgen receptor in hyperplastic prostate and LNCaP cells: implications for chemoprevention of prostate cancer" by Hsieh, et al. Prostate 72: 703–704. 21882213 10.1002/pros.21480

[33] Ing NH (2005) Steroid hormones regulate gene expression posttranscriptionally by altering the stabilities of messenger RNAs. Biol Reprod 72: 1290–1296. 15728791 10.1095/biolreprod.105.040014

[34] Lin MC, Rajfer J, Swerdloff RS, Gonzalez-Cadavid NF (1993) Testosterone down-regulates the levels of androgen receptor mRNA in smooth muscle cells from the rat corpora cavernosa via aromatization to estrogens. J Steroid Biochem Mol Biol 45: 333–343. 8499343 10.1016/0960-0760(93)90002-e

[35] Schmidt LJ, Tindall DJ (2013) Androgen receptor: past, present and future. Curr Drug Targets 14: 401–407. 23565753 10.2174/1389450111314040002

[36] Waltering KK, Helenius MA, Sahu B, Manni V, Linja MJ, et al (2009) Increased expression of androgen receptor sensitizes prostate cancer cells to low levels of androgens. Cancer Res 69: 8141–8149. 19808968 10.1158/0008-5472.CAN-09-0919

[37] Chhipa RR, Halim D, Cheng J, Zhang HY, Mohler JL, et al (2013) The direct inhibitory effect of dutasteride or finasteride on androgen receptor activity is cell line specific. Prostate 73: 1483–1494. 23813737 10.1002/pros.22696 PMC3992475

[38] Banerjee PP, Banerjee S, Brown TR (2001) Increased androgen receptor expression correlates with development of age-dependent, lobe-specific spontaneous hyperplasia of the brown Norway rat prostate. Endocrinology 142: 4066–4075. 11517186 10.1210/endo.142.9.8376

[39] Brand LJ, Dehm SM (2013) Androgen receptor gene rearrangements: new perspectives on prostate cancer progression. Curr Drug Targets 14: 441–449. 23410127 10.2174/1389450111314040005 PMC3957184

[40] Leader JE, Wang C, Fu M, Pestell RG (2006) Epigenetic regulation of nuclear steroid receptors. Biochem Pharmacol 72: 1589–1596. 16844098 10.1016/j.bcp.2006.05.024

[41] Kato S, Yokoyama A, Fujiki R (2011) Nuclear receptor coregulators merge transcriptional coregulation with epigenetic regulation. Trends Biochem Sci 36: 272–281. 21315607 10.1016/j.tibs.2011.01.001

[42] Li J, Al-Azzawi F (2009) Mechanism of androgen receptor action. Maturitas 63: 142–148. 19372015 10.1016/j.maturitas.2009.03.008

[43] Lim AC, Attard G (2013) Improved therapeutic targeting of the androgen receptor: rational drug design improves survival in castration-resistant prostate cancer. Curr Drug Targets 14: 408–419. 23565754 10.2174/1389450111314040003

[44] Nyquist MD, Dehm SM (2013) Interplay between genomic alterations and androgen receptor signaling during prostate cancer development and progression. Horm Cancer 4: 61–69. 23307762 10.1007/s12672-013-0131-4 PMC3957092

[45] Perobelli JE, Patrao MT, Fernandez CD, Sanabria M, Klinefelter GR, et al (2013) Androgen deprivation from pre-puberty to peripuberty interferes in proteins expression in pubertal and adult rat epididymis. Reprod Toxicol 38: 65–71. 23541399 10.1016/j.reprotox.2013.03.004

[46] Bolton EC, So AY, Chaivorapol C, Haqq CM, Li H, et al (2007) Cell- and gene-specific regulation of primary target genes by the androgen receptor. Genes Dev 21: 2005–2017. 17699749 10.1101/gad.1564207 PMC1948856

[47] Isidori AM, Buvat J, Corona G, Goldstein I, Jannini EA, et al (2013) A critical analysis of the role of testosterone in erectile function: from pathophysiology to treatment-A systematic review. Eur Urol 2838: 876–872. 10.1016/j.eururo.2013.08.048 24050791

[48] Traish AM (2009) Androgens play a pivotal role in maintaining penile tissue architecture and erection: a review. J Androl 30: 363–369. 18802199 10.2164/jandrol.108.006007

[49] Zhang MG, Wu W, Zhang CM, Wang XJ, Gao PJ, et al (2012) Effects of oral finasteride on erectile function in a rat model. J Sex Med 9: 1328–1336. 22375859 10.1111/j.1743-6109.2012.02661.x

[50] Pinsky MR, Gur S, Tracey AJ, Harbin A, Hellstrom WJ (2011) The effects of chronic 5-alpha-reductase inhibitor (dutasteride) treatment on rat erectile function. J Sex Med 8: 3066–3074. 21834872 10.1111/j.1743-6109.2011.02425.x

[51] Iacono F, Prezioso D, Ruffo A, Illiano E, Romis L, et al (2012) Testosterone deficiency causes penile fibrosis and organic erectile dysfunction in aging men. Evaluating association among Age, TDS and ED. BMC Surg. 12: S24. 23173727 10.1186/1471-2482-12-S1-S24 PMC3499353

[52] Zhang MG, Wang XJ, Shen ZJ, Gao PJ (2013) Long-term oral administration of 5alpha-reductase inhibitor attenuates erectile function by inhibiting autophagy and promoting apoptosis of smooth muscle cells in corpus cavernosum of aged rats. Urology 82: 743.e749–743.e715. 10.1016/j.urology.2013.02.045 23876578

[53] Geller J (1990) Effect of finasteride, a 5 alpha-reductase inhibitor on prostate tissue androgens and prostate-specific antigen. J Clin Endocrinol Metab 71: 1552–1555. 1699965 10.1210/jcem-71-6-1552

[54] Duskova M, Hill M, Starka L (2010) Changes of metabolic profile in men treated for androgenetic alopecia with 1 mg finasteride. Endocr Regul 44: 3–8. 20151762 10.4149/endo_2010_01_3

[55] Grino PB, Griffin JE, Wilson JD (1990) Testosterone at high concentrations interacts with the human androgen receptor similarly to dihydrotestosterone. Endocrinology 126: 1165–1172. 2298157 10.1210/endo-126-2-1165

[56] Cobb JE, White SJ, Harrap SB, Ellis JA (2009) Androgen receptor copy number variation and androgenetic alopecia: a case-control study. PLoS One 4: e5081. 19340294 10.1371/journal.pone.0005081 PMC2659771

[57] Cecchin E, De Mattia E, Mazzon G, Cauci S, Trombetta C, et al. (2014) A pharmacogenetic survey of Androgen Receptor polymorphisms in patients experiencing long term toxicity after finasteride withdrawal. Int J Biol Markers. May 17. 10.5301/jbm.5000095 24855036

[58] Chang C, Lee SO, Wang RS, Yeh S, Chang TM (2013) Androgen receptor (AR) physiological roles in male and female reproductive systems: lessons learned from AR-knockout mice lacking AR in selective cells. Biol Reprod. 89: : 21,1–16. 10.1095/biolreprod.113.109132 PMC4076350 23782840

[59] Pozzi P, Bendotti C, Simeoni S, Piccioni F, Guerini V, et al (2003) Androgen 5-alpha-reductase type 2 is highly expressed and active in rat spinal cord motor neurones. J Neuroendocrinol 15: 882–887. 12899683 10.1046/j.1365-2826.2003.01074.x

[60] Marron TU, Guerini V, Rusmini P, Sau D, Brevini TA, et al (2005) Androgen-induced neurite outgrowth is mediated by neuritin in motor neurones. J Neurochem 92: 10–20. 15606892 10.1111/j.1471-4159.2004.02836.x

